# Supplementary material for: Patterns of Intron Gain and Loss in Fungi
Source: PLoS Biol. 2004 Nov 30;2(12):e422. doi: 10.1371/journal.pbio.0020422 (PMC532390; doi:10.1371/journal.pbio.0020422)
Supplement: Table S1 — Also available at http://genes.mit.edu/NielsenEtAl/. (4.3 MB ZIP). [file pbio.0020422.st001.zip › NielsenEtAl/html/346.html]

AN6892.1.NCU00434.1.MG05207.1.FG04111.1


```
 CLUSTAL W (1.82) Multiple Sequence Alignments - Introns Inserted


Sequence 1: MG05207.1	561 aa
Sequence 2: FG04111.1	577 aa
Sequence 3: NCU00434.1	619 aa
Sequence 4: AN6892.1	566 aa
Alignment Length: 637 aa
Number Identitical Residues: 237 aa
Alignment Score (without introns) 10730


MG05207.1 	MFGG-SSKADSPTLTSPTSSLPATATPAPGSKSPSPD---ASESTLSSSPTKAAEQATSR
NCU00434.1	MFGGGSSSAASKSVSGSASDSGSSGKDRSSSKSPSPEKEAPGNGGASPKSIKSADQSTAG
FG04111.1 	MFGG-SSNNSAGNKSESDS---------KSDKSPSPE---PGTGLSAPSPIKTTDQAASG
AN6892.1  	MFSGSSSPPKDKSDTIPRS---------GPINTPSLS--IQTEGTVGSQAKSPPPTSFFG
          	**.*.**     . : . *            ::** .      .  .... ...  :   

MG05207.1 	EKRSGNGSPPD--SAGAAEQKKRRSSGVSSKASSLLASAKNTLNFGQGSSRSN---TDNG
NCU00434.1	DTQIGNGSPPS--AGSSSDSKKKRSSGVSGRASSLIAQAKNTLFTQVANRNSGDMSTDSK
FG04111.1 	EKRSGNGSPPTRQDVGGPSDKKRRSSSVSSRASSLLASAKNSLNFSSQSSRGG---GSE-
AN6892.1  	RRASEEQSPGG--------DKKRRSSTVS-KAASFFANAKNSLSLSSPRTESA----LSS
          	     : **          .**:*** ** :*:*::*.***:*       ..      . 

MG05207.1 	SSTQTALQKLGKQDPALVVPQGQQNNSAGESLPGPKSTFRVGVWEDRNKKCRRTMEDTHA
NCU00434.1	NSNQTSLQKLGKQDQALAVPQGQHNNAAGESLPGPRSTFKVGVWEDRNKKCRRTMEDTHA
FG04111.1 	VNSQTSLQKLGKQDPALAVPQGQHNNSAGDSMPGPKSTFRVGVWEDRNKKCRRTMEDTHA
AN6892.1  	SLPESSLHRYGNMDPALNVPQGSLNNSAGESSPTPRSSFRVGVTEDRNKKCRRTMEDTHA
          	   :::*:: *: * ** ****. **:**:* * *:*:*:*** ****************

MG05207.1 	FLYNFLHTPA----------ATPASSSNATDGETKTTATPGDEIVESDNGYFAIFDGHAG
NCU00434.1	FLYNFLHTPAPVLGAEGSAQKQSKSANESHEKRSSSSASQNADMIETDNGYFAIFDGHAG
FG04111.1 	FLYNFVQTPA----------LNDASAKDKPEGDDLVTS----DMVESDNGYFAIFDGHAG
AN6892.1  	YLYNFLGNPVP-------SAQNDDSDQSSHPSDASPKTGELTPIVETDNGYFAIFDGHAG
          	:****: .*..       :     * ..        .:     ::*:*************

MG05207.1 	TFAADWCGKKLHLILEETIRKKPNVPIPELLDETFTTVDAQLEKLPVKNSGCTAAIAVLR
NCU00434.1	TFAADWCGKKLHIILEDIIKKNPNAPIPELLDQTFTTVDTELEALPLKNSGCTAAVAVLR
FG04111.1 	TFAADWCGKKLHIILEDIIRKNPNALIPEVLDQAFTAADAQLEKLPLKNSGCTAAVAVLR
AN6892.1  	TFAAQWCGKKLHLILEDVMRKNINAPVPELLDQTFTSVDQQLEKLPVKNSGCTAVVALLR
          	****:*******:***: ::*: *. :**:**::**:.* :** **:*******.:*:**

MG05207.1 	WEDRVPSSQSATGSQPVAA--AKASDEGLKPEDGKS-NDGASLRASDAAPPRPKPAVTKQ
NCU00434.1	WEDRVPSNQSATGSQAIAPAVVKATEDALKVEDGQS-EKSLNSSIPEATHAKLKNSATRQ
FG04111.1 	WEDRASNDRSAI--KPVKP--EEP------IEMDKA-NDGSSNEEATKATNSKGKGSGRQ
AN6892.1  	WEDRIPTPSSVTGSSAIGPAAVAATKAGADSKADDTPTQEAAATPVPAIPPKLREKAIRQ
          	**** ..  *. .:..: .:   .:. . . : ..:. .                   :*

MG05207.1 	RVLYTANVGDARIILCRSGKALRLSYDHKGSDENEGRRITNAGGLILNNRVNGVLAVTRA
NCU00434.1	RVLYTANVGDARIVLCRSGKALRLSYDHKGSDENEGKRIANAGGLILNNRVNGVLAVTRA
FG04111.1 	RVLYTANVGDARIILCRGGKALRLSYDHKGSDEVEGKRIAAAGGLILNNRVNGVLAVTRA
AN6892.1  	RVLYTANVGDARVILCRNGKALRLSYDHKGSDENEGRRIANAGGLILNNRVNGVLAVTRA
          	************::***.*************** **:**: *******************

MG05207.1 	LGDTYMKELVTGHPYTTETVIQPELDEFLIIACDG0LWDVCDDQEAVDQVRNIEDPAAAA
NCU00434.1	LGDTYIKDLVTGHPYTTETVIQPDWDEFMIIACDG0LWDVCSDQEAVDLVRDIQEPVIAA
FG04111.1 	LGDAYMKKLVTGHPYTTETVIQPDSDEFIIIACDG0IWDVCSDQEAVDLVRNVNDPISAS
AN6892.1  	LGDAYIKDLVTGHPYTTETVIQPDLDEFIILACDG0LWDVCSDQEAVDLIRNVSDAQEAS
          	***:*:*.***************: ***:*:**** :****.****** :*::.:.  *:

MG05207.1 	KLLVNHALARFSTDNLSCMIVRFD--KQAVLDGHSRKD--------------AATKGD--
NCU00434.1	KKLVDHALSRFSTDNLSCMIVRFD--KQALLENQNNKDSAIGVEGDATTAAGTVTESEKI
FG04111.1 	KQLVDYALNRFSTDNLSCMVVRLDQNKEAAVEVEGAAS--------------QVSEADKI
AN6892.1  	KILVDHALARFSTDNLSCMVIRLYADRHREVASQAVDLAG------------WESRSSRG
          	* **::** **********::*:  .:.  :  .    :.              :...  

MG05207.1 	-------------PASKPSEA--DRNVQLATNAAGRSNDPIPS-----ISEEP-------
NCU00434.1	VTATKQKIAEGSTPAVGISASNFGRGHDPTPLEDGEKKPFVPTTIEGSVEEEP-------
FG04111.1 	VSETKQKIAEGIAPAVGVSAT--SNAPQSQPPIAVQEGDFVPTSLDDIVTEEPPHVADPK
AN6892.1  	LSET-----DKIVEGARKSMANTGIADESETLKKVHDEAEECTP-----EEVP-------
          	 : :     .    .   * :. .   :  .    ..     :.      * *       

MG05207.1 	--------AAAAAAAAGDSSGGTKGQDDETSQPATSS-
NCU00434.1	------VVLSDSPEATPDGSVTAMKLDSLPEAKKSLDA
FG04111.1 	SDDVPILNKAAVLEARKDKPAEEKPVEEQETPPGQE--
AN6892.1  	---------ESILSASPNAPAATLNQPNKPGSSDST--
          	              *  : .       .
```
